# Outcomes of initial 100 cases of laparoscopic nephrectomy at tertiary care center (PKLI)

**DOI:** 10.12669/pjms.40.1.7819

**Published:** 2024

**Authors:** Nadeem Iqbal, Syed Imtiaz Ali, Ayesha Farooq, Nauman Zafar

**Affiliations:** 1 Nadeem Iqbal, Pakistan Kidney and Liver Institute, Lahore, Pakistan; 2 Syed Imtiaz Ali, Medcare Hospital Jumairah, Dubai, UAE; 3 Ayesha Farooq, Pakistan Kidney and Liver Institute, Lahore, Pakistan; 4 Nauman Zafar, Pakistan Kidney and Liver Institute, Lahore, Pakistan

**Keywords:** Laparoscopic nephrectomy, Complication, Clavien classification system

## Abstract

**Objective:**

Laparoscopic nephrectomy has been adopted by many centers in the last few decades. However, there are a few inherent challenges while adopting laparoscopic nephrectomy in a new unit. These include a significant learning curve required to adopt this modality. This study aimed to share the initial experience of adopting laparoscopic nephrectomy at our center.

**Methods:**

In total, 101 patients were analyzed in the study. These patients underwent laparoscopic radical or simple nephrectomy (for renal mass and noncancer renal cases respectively) at Department of Urology, Pakistan Kidney and Liver Institute and Research Centre, Lahore from April 2018 till January 2021. Data were entered in the statistical analysis software file. Analysis was attained by utilizing SPSS version 20. Implementation of Mean along with standard deviation values was utilized in the case of the continuous variables. While frequency/percentages represented categorical factors.

**Results:**

The mean age of patients was 42.81±15.49 years and their overall BMI was 26.41±5.30 kg/m^2^. Out of these, 57 (56.43%) were males and 44(43.56%) were female. Eighteen percent of patients had a previous surgical history on the ipsilateral side. Total operative time was 163.98±58.02 minutes while mean hospital stay reached 3.2±0.87 days. The tumor-free margin was attained in all cases of radical nephrectomy. Based on Clavien-Dindo classification, Grade-1 (n=3; 2.97%), Grade-2 (n=6; 5.94%), Grade-3A (n=1; 0.99%), and Grade-3B (n=1; 0.99%) complications were observed.

**Conclusion:**

In a newly developed urology center, laparoscopic nephrectomy can be a daunting task. Good teamwork among the surgical team members and careful selection of cases can result in satisfactory procedural outcomes.

## INTRODUCTION

Clayman et al. mentioned the first laparoscopic nephrectomy performed for a renal mass.^1^ Later on, there was a spread of interest in laparoscopic nephrectomy owing to its minimally invasive nature. More urologists embarked on laparoscopic procedures as their confidence and skill levels enhanced over time. As compared to other procedures in Urology, laparoscopic nephrectomy was easier to adopt, resulting in its popularity among urologists.^2^ Many studies were shared by urologists who practiced both laparoscopic surgeries and the open approach. Literature proved laparoscopic nephrectomy to be a better option when compared to open nephrectomy. It was associated with lesser analgesic requirements in the post-operative recovery period, a swift return to the daily routine, lower complications rate, and shorter hospital stays.^3^-^7^

However, there are a few inherent challenges while adopting laparoscopic nephrectomy in a new unit. These include a significant learning curve required to adopt this modality. Additionally, the new setup and establishment of the operation theatre to embark on laparoscopic procedures, proper training of staff and technicians, and proper care and use of instruments.^8^ This study aimed to share the initial experience of adopting laparoscopic nephrectomy at our center. We focused on outcomes such as the successful completion of the procedure and related complications.

## METHODS

In total, 101 patients were analyzed in the study. These patients underwent laparoscopic radical or simple nephrectomy (for renal mass and noncancer renal cases respectively) at our department from April 2018 till January 2021. The study aimed to look for initial experience regarding surgical outcomes of laparoscopic nephrectomy, in regards to their total operative time, need for conversion to open surgery, tumor-free margins (in renal cancer cases), postoperative complications, analgesic requirements, and hospital stay. Informed consent was acquired from all patients prior to the procedure and they were counseled regarding possible outcomes and complications of the procedure.

### Ethical Approval

For undertaking this retrospective study, we acquired approval from the ethical committee at our hospital (IRB no PKLI-IRB/AP/77).

### Exclusion Criteria

Patients of age less than 18 years, those having abnormal coagulation profiles, those not willing to undergo the procedure, subjects having congenital renal anomaly, and those who failed to come for follow-up were excluded from the study. Patient demographic information was recorded by registrars at the department, which included patient age, gender, past surgical history for stone disease, and body mass index (BMI). CT scans with contrast images were assessed in all renal cancer patients to elaborate on the surgical anatomy and planning of the procedure. Pre and Post-operative variables were recorded comprising of the side of surgery, indication for surgery, hemoglobin drop after the procedure, need for blood transfusion, demand for the analgesics, recording of procedure-related complications (modified clavian classification), and date of discharge from the hospital.

Once decision of doing laparoscopic nephrectomy was pursued, blood investigations consisting of complete blood count, renal functions (serum urea, blood urea nitrogen and creatinine), electrolytes and coagulation tests, blood grouping and cross matching were done prior to the time of inpatient admission. One dose of Injection ceftriaxone was given at the induction of anesthesia. These laparoscopic surgeries were performed by a single surgeon. The transperitoneal approach was used in laparoscopic nephrectomy. The patient was placed in the flank position. Three trocars were used for the procedure. The first trocar (12mm) was placed lateral to the rectus sheath at the umbilical level. The pneumoperitoneum was achieved using the open Hasson technique. The second and third trocars (one 11mm and another 5mm) were placed at the mid-axillary line in such a way that the triangle was made.

The colon was reflected medially until Gerota’s fat and the psoas muscle were identified. Along the psoas muscle, the ureter was identified and lifted in a sling with the help of a needle placed under vision at the mid-axillary line. The ureter was followed proximally; the kidney was mobilized up to the hilum. The ureter was clipped and incised first to help mobilize the kidney from the posterior side. Hem-o-Lok clips were applied to fasten the renal artery and vein before dividing them. Then, the specimen was retrieved with the help of an organ retrieval bag.

### Statistical Analysis

Data was gathered in the proformas by the urology registrar, it was entered in the statistical analysis software file. Analysis was attained by utilizing SPSS version 20. Implementation of Mean along with standard deviation values was utilized in the case of the continuous variables. While frequency/percentages represented categorical factors.

## RESULTS

A total of 101 patients were analyzed in this retrospective study. Their mean age was 42.81±15.49 years and their overall BMI was 26.41±5.30 kg/m^2^. Out of these, 57 (56.43%) were males and 44(43.56%) were female. Eighteen percent of patients had a previous surgical history on the ipsilateral side. These previous surgeries included open stone surgeries or pyeloplasties. The mean ASA score was 1.6±0.59.

The majority of the patients (n=52, 51.48%) had laparoscopic nephrectomy on the right side while 49 (48.51%) had it on the left side. In total, 41(40.59%) of these patients had surgery for kidney mass (radical nephrectomy) while the remaining cases were simple nephrectomies. Total operative time was 163.98±58.02 minutes while mean hospital stay reached 3.2±0.87 days. Their preoperative hemoglobin was 13.2±2.97 g/dl while the post-operative hemoglobin diminished to 11.2±1.83 g/dl. Tumor-free margin was attained in all cases of radical nephrectomy.

We had to convert seven cases to open surgery because of difficulty in the progression of the laparoscopic surgery or bleeding obscuring the view. There was one case of iatrogenic duodenal injury needing repair. Post-wound infection was seen in one case. Based on Clavien-Dindo classification, Grade-1 (n=3; 2.97%), Grade-2 (n=6; 5.94%), Grade-3A (n=1; 0.99%), and Grade-3B (n=1; 0.99%) complications were observed (Table-II).

**Table-I:** Demographic variables.

Variable	Value
Number	101
Mean Age	42.81±15.49 years
Male	57 (56.43%)
Female	44(43.56%)
Right side procedure	52 (51.48%)
Left side procedure	49 (48.51%)
Body Mass Index	26.41±5.30 kg/m^2^
Radical nephrectomy	41(40.59%)
Simple nephrectomy	60 (59.41%)

**Table-II:** Details of Procedure Outcomes.

Variables	Values
Tumor free margin	41/41 (100%)
Operative time	163.98±58.02 minutes
Hospital Stay	3.2±0.87 days
Analgesic doses	2.72±1.56
Drop in haemoglobin	2.21±0.73
***Complications***	
Illeus (Grade-1)	2 (1.98%)
Fever (non-infectious) (Grade-1)	1 (0.99%)
Transfusion (grade-2)	3 (2.97%)
Wound infection (Grade-2)	2 (1.98%)
Pneumonia (Grade-2)	1 (0.99%)
Subphrenic abscess requiring drainage (Grade 3-A)	1 (0.99%)
Duodenal injury (Grade 3-B)	1 (0.99%)

## DISCUSSION

Laparoscopic surgery has progressed much over the past two decades. There have been improvements in Techniques and instruments that have paved the way to bring a progressive paradigm shift from traditional open surgery toward minimally invasive surgery. This mindset change has also been adopted in the case of the management of genitourinary oncologic conditions. This transition has gained significant momentum due to the increasing number of educated patients who desire less traumatic and more cosmetic approaches to their diseases. With the passage of time, there has been technological progress to enhance vision and innovation in making sophisticated and surgeon-friendly instruments: all of which have boosted the confidence of urologists to offer successful undertaking of even complex and delicate reconstructive procedures. Even in cases of Xanthogranulomatous pyelonephritis, laparoscopic nephrectomy has been tried successfully in well selected patients. Recently, Laparoendoscopic single-site donor nephrectomy was performed safely even in patients with duplicated inferior vena cava.^9^-^12^

The term laparoscopy is now frequently utilized to explain both intraperitoneal and extraperitoneal endoscopic procedures. For keeping procedures more specific, terms such as transperitoneal and retroperitoneal laparoscopy are used. This modality is adopted to deal with both cancer and non-cancer renal cases. Both simple and Radical nephrectomy can be executed either purely laparoscopically (using only trocars) or hand-assisted (wherein an added incision of approximately 4-inch length is given together with two to three trocars via a transperitoneal approach). Strict adherence to the principles of surgical oncology improves the laparoscopic surgery outcome comparable to that achieved in open surgery. Laparoscopy offers less morbidity, improved operative precision and better cosmesis.^11^-^14^ Several studies have reported shorter periods required for a return to full activity (three to four weeks) in contrast to 8 to 10 weeks that may be taken after open surgery.^13^-^16^

Mithani et al from Karachi shared their experience with initial 100 cases of laparoscopic nephrectomy. The mean age in their cohort was 34.1 ± 15.1 years. Fifty-four percent of their cases were right-sided. Thirty-two percent of their cases had a prior history of abdominal surgery, such as appendectomy, C-section, or pyelolithotomy.^14^ In the present study, 51.48% had laparoscopic nephrectomy on the right side and eighteen percent of the patients had a previous surgical history on the ipsilateral side.

Quddus et al. from a training institute shared their initial experience with laparoscopy in 36 patients. They had to convert to open surgery in 8 cases (22.2%), a higher conversion rate.^15^ In the present study, we had to convert seven cases to open surgery because of difficulty in the progression of the laparoscopic surgery or bleeding obscuring the view. Mithani et al.^14^ reported an operative time reaching 108 minutes, and Zaidi et al.^16^ shared their experience in performing laparoscopic nephrectomies in 60 patients with a mean operative time reaching 140 + 51.1 minutes. Quddus et al had a mean operative time of 216 ± 100 minutes.^15^ while the operative time in our patients was 163.98±58.2 minutes.

Mithani et al had Very few complications; one percent of wound infections, one percent of prolonged ileus, while two percent cases required blood transfusion.^14^ Similarly, Quddus et al reported majority complications being Clavien Grade-2 including 10% blood transfusion.^15^ We categorized complications based on Clavien-Dindo classification: Grade 1 (n=3; 2.97%), Grade-2 (n=6; 5.94%), Grade-3A (n=1; 0.99%), and grade 3B (n=1; 0.99%) complications were observed (Table-II). Balcı et al. reported Grade 1 (1.4%), 2 (4.3%), and 3A (0.5%) complications in their study.^17^ One case (0.5%) needed simultaneous splenectomy owing to iatrogenic splenic rupture. In the present study, one case of iatrogenic duodenal injury was encountered that was repaired.

Balcı et al. shared their experience with 208 patients having mean age was 48.01±14.9 years and a mean duration of hospital stay was 3.5±1.9 days.^17^ Campos et al. reported a prolonged operative time of 199.3±61 minutes and a hospital stay of 5.7±3 days.^18^ In the present study, the mean hospital stay reached 3.2±0.87 days. This was slightly lesser as compared to the study shared by Balcı et al. Another study by Xu et al. reported a complication rate of 19.31% in the laparoscopic radical nephrectomy group. They noted more Grade II complications and prolonged postoperative hospital stay in the open radical nephrectomy group in contrast to the laparoscopic radical nephrectomy.^19^

We faced challenges on multi steps and they were sorted out gradually. We have summarized and explained concisely in Table-III these factors of challenges and learning curve and how we progressed through these challenges.

**Table-III:** Details of Procedure Learning Curve Progress.

Variables of selection and progress	First 50 cases	Next 50 cases
ASA	ASA1=35	ASA1=15
	ASA2=13	ASA2=28
	ASA3=2	ASA3=8
Learning Curve under supervision of Mentors (cases done)	Holding camera=5 cases	Complete procedure Mentor scrubbed=21 cases.
	Ports insertion + Colon mobilization=11 cases	
	Ureter Mobilization=13 cases	Complete procedure Mentor un scrubbed but in theatre=24 cases
	Renal hilum dissection=15 cases	Complete procedure Mentor not in theatre except at time of last clipping of hilum vessels and specimen removal=6 cases.
	Renal vessels clipping and specimen removal=6 cases	
Hospital stay	3.96±0.57 days	2.51±0.73 days
Operative time	197.71±33.76 minutes	143.82±28.36 minutes
HDU stay	2.41±0.3 days	1.09±0.4 days
Frequency procedure per week	Initial 10 cases=0.5 case per week	Two cases per week
	Next 40 cases=1 case per week	
Complexity of case	Simple nephrectomy=39	Simple nephrectomy=21
	Radical nephrectomy=11	Radical nephrectomy=30
Conversion to open	5	2

### Limitations

It was a retrospective single-center study and the sample size was not a large one. Further studies regarding the learning difficulties in undertaking laparoscopic nephrectomies at new centers are needed in the future in order to find the challenges and their possible solutions.

## CONCLUSION

In a newly developed urology center, laparoscopic nephrectomy can be a daunting task. Good teamwork among the surgical team members and careful selection of cases can result in satisfactory outcomes in terms of the success of the procedure and acceptable complication rates.

### Authors’ Contributions:

**NI**, **IA** and **NZ:** Conceived, designed, did statistical analysis & editing of the manuscript, is responsible for the integrity of research.

**NI**, **IA**, **NZ** and **AF:** Did data collection and manuscript writing.

**NI**, **AF** and **IA:** Did review and final approval of the manuscript.

**NI**: Responsible for the accuracy or integrity of the work.
